# The Immunocytokine FAP-IL-2v Enhances Anti-Neuroblastoma Efficacy of the Anti-GD_2_ Antibody Dinutuximab Beta

**DOI:** 10.3390/cancers14194842

**Published:** 2022-10-04

**Authors:** Nikolai Siebert, Justus Leopold, Maxi Zumpe, Sascha Troschke-Meurer, Simon Biskupski, Alexander Zikoridse, Holger N. Lode

**Affiliations:** Department of Pediatric Oncology and Hematology, University Medicine Greifswald, 17475 Greifswald, Germany

**Keywords:** neuroblastoma, immunotherapy, dinutuximab beta, fibroblast activation protein α, FAP-IL-2v, myeloid-derived suppressor cells

## Abstract

**Simple Summary:**

Since IL-2 co-treatment did not show any therapeutic benefit in the GD_2_-directed treatment of high-risk neuroblastoma (NB) but strongly induced regulatory T cells (Treg), we investigated here the immunocytokine FAP-IL-2v stimulating NK and cytotoxic T cells without induction of Treg. We first detected FAP on NB- and myeloid-derived suppressor cells (MDCS) in tumor tissue and showed a tumor-cell-dependent enhancement in FAP expression by fibroblasts. Treatment of leukocytes with FAP-IL-2v increased ADCC mediated by the anti-GD_2_ antibody dinutuximab beta (DB) against NB cells. We next evaluated the antitumor efficacy of a combinatorial immunotherapy by applying DB and FAP-IL-2v and observed strongly reduced tumor growth and improved survival in experimental mice. Analysis of tumor tissue revealed increased NK and cytotoxic T cell numbers and reduced Treg compared to controls. Our data show that FAP-IL-2v is a potent immunocytokine that augments the efficacy of DB against NB, providing a promising alternative to IL-2.

**Abstract:**

Treatment of high-risk neuroblastoma (NB) patients with the anti-GD_2_ antibody (Ab) dinutuximab beta (DB) improves survival by 15%. Ab-dependent cellular cytotoxicity (ADCC) is the major mechanism of action and is primarily mediated by NK cells. Since IL-2 co-treatment did not show a therapeutic benefit but strongly induced Treg, we investigated here a DB-based immunotherapy combined with the immunocytokine FAP-IL-2v, which comprises a fibroblast activation protein α (FAP)-specific Ab linked to a mutated IL-2 variant (IL-2v) with abolished binding to the high-affinity IL-2 receptor, thus stimulating NK cells without induction of Treg. Effects of FAP-IL-2v on NK cells, Treg and ADCC mediated by DB, as well as FAP expression in NB, were investigated by flow cytometry, calcein-AM-based cytotoxicity assay and RT-PCR analysis. Moreover, the impact of soluble factors released from tumor cells on FAP expression by primary fibroblasts was assessed. Finally, a combined immunotherapy with DB and FAP-IL-2v was evaluated using a resistant syngeneic murine NB model. Incubation of leukocytes with FAP-IL-2v enhanced DB-specific ADCC without induction of Treg. FAP expression on NB cells and myeloid-derived suppressor cells (MDCS) in tumor tissue was identified. A tumor-cell-dependent enhancement in FAP expression by primary fibroblasts was demonstrated. Combination with DB and FAP-IL-2v resulted in reduced tumor growth and improved survival. Analysis of tumor tissue revealed increased NK and cytotoxic T cell numbers and reduced Treg compared to controls. Our data show that FAP-IL-2v is a potent immunocytokine that augments the efficacy of DB against NB, providing a promising alternative to IL-2.

## 1. Introduction

Neuroblastoma (NB) is a malignant disease of childhood with a poor prognosis in the high-risk group [1]. In Europe, treatment of high-risk NB patients with the chimeric anti-GD_2_ antibody (Ab) dinutuximab beta (DB) in combination with the immune stimulating cytokine interleukin-2 (IL-2) resulted in a 15% improvement in 5-year survival compared to the standard treatment [2]. Therapeutic Ab directed against tumor antigens mediates antitumor effects primarily through the induction of antibody-dependent cellular cytotoxicity (ADCC), whereby natural killer (NK) cells are the major effector cells. Indeed, it was shown that the depletion of NK cells resulted in the complete abrogation of the antitumor effects mediated by DB [3]. The rationale of combining DB with IL-2 for NB treatment was based on the stimulating effects of IL-2 on NK cells. However, evaluation of the progression-free (PFS) and overall survival (OS) probabilities in high-risk NB patients treated with or without IL-2 did not reveal any additional treatment benefit of IL-2, while higher treatment-related toxicity was observed in the patients who additionally received IL-2 [4]. Although administration of IL-2 resulted in an approximately three-fold increase in cytotoxic NK cells [5], thus confirming the rationale of using IL-2 in combination with DB, the undesired strong expansion of regulatory T cells (Treg) with increased levels over 20-fold compared to the baseline was also detected in the patients of the combinatorial cohort [5]. These results explain in part the unexpected absence of the clinical benefit of IL-2.

The use of alternative immune-stimulating agents that do not preferentially induce immune-inhibiting cells may provide an alternative. One promising molecule in this context is the mutated variant of IL-2 (IL-2v), with reduced binding to the IL-2RA subunit of the high-affinity trimeric IL-2 receptor (IL-2RABG; A, B and G for α (CD25), β (CD122) and γ receptor chain (CD132)) [6] expressed by Treg but with efficient binding to the intermediate-affinity dimeric IL-2RBG [6] expressed by NK and resting T cells. Recently, the proliferation of NK cells and effective activation of cytotoxic T cells without preferential activation of Treg has been shown in vitro after incubation of peripheral blood mononuclear cells with IL-2v conjugated with an Ab against the fibroblast activation protein α (FAP) [7]. Due to abolished Fcγ receptor binding (P329G LALA mutations), FAP-IL-2v does not induce ADCC against FAP-positive cells and serves as a vehicle for IL-2v, transporting it into the tumor microenvironment. Importantly, the activating effects of FAP-IL-2v on effector cells by IL-2v has been shown to be translated into the considerably enhanced antitumor activity of these effector cells against tumor cells mediated by the therapeutic Ab [7], thus clearly showing advantageous therapeutic effects compared to the non-modified IL-2.

Moreover, as the administration of cytokines is known to be associated with systemic side effects, the usage of immunocytokines (tumor-specific Ab genetically linked to cytokines) transporting cytokines directly into the site of tumor could overcome this obstacle. A common tumor-associated antigen is the fibroblast-activating protein (FAP), which is detectable in tumor tissue of different malignancies. FAP is a dimeric protease localized primarily on the cell surfaces of cancer-associated fibroblasts (CAFs), which have been shown to play a protumoral role [8]. Generally, CAFs are known to increase tumor cell invasion, angiogenesis and tumor growth and their presence correlates with a poor prognosis [8]. Importantly, CAFs have been detected in NB [9], thus suggesting FAP targeting as a promising therapeutic strategy against this aggressive tumor. Based on these observations, we hypothesized that a combinatorial treatment of NB with DB and the immunocytokine FAP-IL-2v can further augment the antitumor efficacy of DB.

In the present study, we first investigated the effects of FAP-IL-2v on Treg and ADCC mediated by DB against NB cells. Next, we assessed FAP expression by primary and well-known NB cells, as well as in a murine NB tumor tissue. We then investigated the impact of soluble factors released from tumor cells on FAP expression by primary fibroblasts isolated from murine skin tissue. Finally, we evaluated the antitumor efficacy of the combinatorial treatment with DB and FAP-IL-2v in vivo using our syngeneic murine-resistant NB model, followed by the analysis of tumor-infiltrating effector cells.

## 2. Materials and Methods

### 2.1. Ethics Statement

All procedures involving human participants were in accordance with the ethical standards of the institutional and national research committee and with the 1964 Helsinki declaration and its later amendments or comparable ethical standards. Informed consent was obtained from all individual participants (Ethics Board of the Medical Faculty of the University Greifswald, approval code number: BB 014/14, 24 January 2014). All procedures involving animal experiments were approved by the animal welfare committee (Landesamt für Landwirtschaft, Lebensmittelsicherheit und Fischerei Mecklenburg-Vorpommern, approval code number: LALLF M-V/7221.3-1-011/20, 7 September 2020) and approved and supervised by the commissioner for animal welfare at the University Medicine Greifswald representing the Institutional Animal Care and Use Committee (IACUC).

### 2.2. Cell Cultivation

The human NB cell lines CHLA-20, CHLA-90, CHLA-136 and CHLA-172, as well as in-house-established cell lines from tumor samples derived from high-risk NB patients (HGW-1, HGW-3, HGW-5) [10] and the newly established cell line HGW-B, were cultivated in IMDM (PAN-Biotech GmbH, Aidenbach, Germany) supplemented with 4 mM stable glutamine (Fisher Scientific, Waltham, MA, USA), 30 U/mL penicillin and 0.03 mg/mL streptomycin (0.3× P/S; PAN-Biotech GmbH, Aidenbach, Germany) and 20% FBS Good (PAN-Biotech GmbH, Aidenbach, Germany). The primary cell line HGW-B was established as described for the cell lines HGW-1, HGW-3, HGW-5 [10]. The human NB cells Kelly, SMS-KCN and LAN-1 were cultivated in RPMI 1640 (Capricorn Scientific GmbH, Ebsdorfergrund, Germany) supplemented with 2 mM stable glutamine, 0.3× P/S and 10% Sera Plus (PAN-Biotech GmbH, Aidenbach, Germany). The human FAP-positive cell line Wi-38 served as a positive control was cultivated in DMEM (Capricorn Scientific GmbH, Ebsdorfergrund, Germany) supplemented with 2 mM stable glutamine, 0.3× P/S, 15% FBS Good and 1× NEAA (Capricorn Scientific GmbH, Ebsdorfergrund, Germany). The murine NB cells NXS2-HGW [11,12] used for the tumor cell implantation in vivo were cultivated in DMEM supplemented with 2 mM stable glutamine, 0.3× P/S and 10% FBS Good. Primary adult murine fibroblasts (PAMF) isolated from skin tissue of A/J mice were cultivated in DMEM supplemented with 4 mM stable glutamine, 0.3× P/S, 1× ITS (Capricorn Scientific GmbH, Ebsdorfergrund, Germany) and 15% FBS Good. Prior to cultivation, mycoplasma contamination analysis was performed for every cell line using the MYCOALERT Detection Kit (Lonza Cologne GmbH, Cologne, Germany). Only mycoplasma-negative cell lines were used for experiments. All cell lines were passaged no more than 30 times.

### 2.3. ADCC

To analyze effects of FAP-IL-2v on the cellular cytotoxic activity of effector cells (ADCC) mediated by DB, a non-radioactive calcein-AM-based cytotoxicity assay was used, as previously described [10]. Briefly, leukocytes of healthy donors were cultivated for five days using RPMI 1640 (Capricorn Scientific GmbH, Ebsdorfergrund, Germany) supplemented with 2 mM stable glutamine, 0.3× P/S and 10% Sera Plus (PAN-Biotech GmbH, Aidenbach, Germany). To show the effects of FAP-IL-2v on ADCC, culture medium was supplemented with 1 µg/mL/day of FAP-IL-2v. Untreated leukocytes and leukocytes incubated with IL-2 (3000 IU/mL/day) served as controls. To induce ADCC against NB cells, DB (10 µg/mL) and an effector-to-target cell ratio of 40:1 were used. The GD_2_-positive human NB cells LAN-1 (5000 cells/well) served as targets cells. The GD_2_ specificity of ADCC was confirmed using the anti-idiotype Ab ganglidiomab [13]. The DB-independent cytotoxicity of leukocytes (AICC, antibody-independent cellular cytotoxicity) was evaluated by incubation of leukocytes with tumor cells with rituximab.

### 2.4. Isolation of PAMF

To assess FAP expression by primary fibroblasts and address whether the injection of syngeneic NB cells in combination with primary fibroblasts results in the development of CAF-positive tumors, we first isolated fibroblasts from the skin tissue of female adult A/J mice. After mice were sacrificed, the fur was removed with hair removal cream. Following disinfection (70% ethanol), skin samples were extracted and cut into small pieces of around 2 × 2 mm size using a scalpel. Tissue samples were then enzymatically digested using a Tumor Dissociation Kit (Miltenyi Biotech, Bergisch Gladbach, Germany) for 90 min at 37 °C. To remove debris, the samples were then filtered using a 70 µm cell strainer. The obtained single-cell solution was finally cultivated in PAMF-specific medium, as described above.

### 2.5. Analysis of Tumor-Cell-Dependent Impact of FAP Expression by PAMF

To evaluate the effects of soluble factors released by NB cells on the FAP expression on primary fibroblasts, PAMF were cultivated with tumor-conditioned medium (TCM) followed by flow cytometry analysis of FAP. For this, 1 × 10^4^ PAMF of the first or second passage was seeded and cultivated for 24 h. Thereafter, 50% of the culture medium was replaced with the tumor-conditioned medium harvested after 48 h cultivation of the murine NB cells NXS2-HGW.

Finally, the treated cells were harvested and used for flow cytometry analysis.

### 2.6. Establishment of a Resistant Version of the NB Model In Vivo

Since the immunotherapy with DB (i.p., five consecutive days, 3 mg/kg bw/day, start of treatment: four days after tumor cell implantation) showed strong antitumor efficacy against NB in our syngeneic tumor model, resulting in constant tumor regression [12,14], we aimed to establish a more resistant version of this model allowing the evaluation of different therapeutic agents in combination with DB. For this, we started DB treatments in a later tumor growth phase. The new treatment protocol was established by the comparison of the following three time points at which the DB immunotherapy was started: day 11, 12 and 14. Briefly, for all in vivo experiments, mice were randomized prior to tumor cell injection. Female 11-week-old A/J mice (Charles River Laboratories, Sulzfeld, Germany) were granted a two-week acclimatization time, accommodated in groups of maximum 6 animals in standard animal laboratories (12 h light/dark cycle, 20 °C ± 2 °C room temperature, 60% ± 20% humidity) with ad libitum access to water and standard laboratory chow. Mice of all experimental groups were subcutaneously injected with 2 × 10^6^ tumor cells on the left ventral flank, followed by the DB immunotherapy (i.p., five consecutive days, 3 mg/kg/day bw). Untreated controls received an equivalent volume of 0.9% NaCl. The treatments were started either on day 8 or day 11 or day 14 after tumor cell implantation. Tumor and/or treatment burden parameters [12] were assessed every two days after tumor cell injection and daily starting on day 8. Tumors were measured using a caliper, followed by tumor volume calculation according to the formula (length × width × height)/2. Mice were sacrificed when tumors exceeded 750 mm^3^. For those mice killed ahead of schedule, the tumor volume data of the last measurement were included into the calculation of the group-specific average volumes at the subsequent time points.

After a more resistant version of our syngeneic tumor model was established, the new treatment protocol was used in the following in vivo experiments for the evaluation of the antitumor efficacy of the combinatorial therapy with FAP-IL-2v and DB.

### 2.7. Induction of CAF Development in Tumor Tissue In Vivo

Prior to the evaluation of the antitumor efficacy of the combinatorial treatment, we investigated whether injection of the tumor cells NXS2-HGW in combination with the syngeneic PAMF led to the development of CAFs in tumor tissue. For this, mice of an additional experimental group were subcutaneously injected in the left ventral flank with 2 × 10^6^ tumor cells in combination with 1 × 10^6^ PAMF (≥95% viability). Prior to this, PAMF were isolated from murine skin tissue and cultivated for one week, as described above.

### 2.8. Evaluation of Antitumor Effects of Combinatorial Immunotherapy with DB and FAP-IL-2v

To evaluate the treatment efficacy of DB in combination with FAP-IL-2v, a lethal syngeneic murine NB model in a more resistant version was used, as described above. Mice were treated i.p. with DB (five consecutive days, 3 mg/kg/day bw) or FAP-IL-2v Ab (twice a week, 1 mg/kg bw) or with a combination of both. Untreated controls received the equivalent volumes of 0.9% NaCl. Moreover, to show IL-2-dependent effects, mice of an additional control group were treated with IL-2 (twice a week, 3 × 10^6^ IU/kg bw/day).

### 2.9. Flow Cytometry

To identify different populations of tumor-infiltrating leukocytes or fibroblasts or to determine the surface abundance of FAP in different cell types, flow cytometry analysis was performed. For this, PE-labeled anti-human anti-FAP Ab (R&D Systems, Minneapolis, MN, USA) or rabbit anti human/mouse anti-FAP (Abcam plc, Cambridge, UK) and respective PE-labeled Fc-specific secondary Ab (Abcam plc, Cambridge, UK) were used. NB cells were detected using the Alexa Fluor647-labeled anti-GD_2_ chimeric Ab DB. Fibroblasts were identified using FITC-labeled anti-mouse anti-CD140a REAfinity Ab (Miltenyi Biotec, Teterow, Germany). For detection of different tumor-infiltrating leukocyte populations described below, the following Ab were utilized: APC-Vio^®^ 770-labeled anti-mouse CD3 REAfinity™ Ab, VioGreen™-labeled anti-mouse CD4 Antibody REAfinity™, PerCP-labeled anti-mouse CD8a REAfinity™ Ab, Vio^®^ Bright FITC-labeled anti-mouse CD11b REAfinity™ Ab, PE-Vio^®^ 770-labeled anti-mouse CD25 REAfinity™ Ab, PE-labeled anti-mouse CD335 REAfinity™ Ab, APC-labeled anti-mouse FoxP3 REAfinity™ Ab (all Miltenyi Biotec, Teterow, Germany), APC/Cy-7-labeled anti-mouse Ly6C Ab and PerCP-labeled anti-mouse Ly6G Ab (BioLegend, San Diego, CA, USA). For every primary Ab, respective ITC were used to determine the potential background caused by nonspecific Ab binding.

First, primary tumors were resected from the experimental mice, followed by the preparation of tumor tissue single-cell suspensions using a Tumor Dissociation Kit (Miltenyi Biotech, Teterow, Germany), according to the manufacturer’s protocol. After assessment of cell numbers and viability, 1 ∓ 2 × 10^6^ cells were used for the analysis.

The following leukocyte populations were detected in murine tumor tissue using antigen-specific Ab: T cells (CD45+/CD3+), cytotoxic T cells (CD45+/CD3+/CD8+), NK cells (CD45+/CD3−/CD335+), Treg (CD3+/CD4+/CD25+/FoxP3+), CD11b-positive immune cells (CD45+/CD11b+), M-MDSC (CD45+/CD11b+/Ly6C^high^/Ly6G−), PMN-MDSC (CD45+/CD11b+/Ly6C^low^/Ly6G+) and Ly6C− and Ly6G-negative immune cells expressing CD11b (CD45+/CD11b+/Ly6C−/Ly6G−). Respective human leukocyte populations were identified as follows: NK cells (CD3−/CD56+), Treg (CD3+/CD4+/CD25+/CD127−).

To exclude unspecific binding of the detection Ab to Fc receptor-expressing cells, samples were first incubated with the FcR Blocking Reagent (Miltenyi Biotech, Teterow, Germany). For intracellular staining, the FoxP3 Staining Buffer Set (Miltenyi Biotech, Teterow, Germany) was used according to the manufacturer’s protocol. To exclude dead cells in the samples prepared for the detection of the cell surface antigens, 4 μL of a 0.1 mg/mL 4′,6-diamino-2-phenylindole (DAPI) solution was added 5 min prior to acquisition using a BD CANTO II cytometer and FACS Diva software (BD Biosciences, Heidelberg, Germany). For the intracellular staining, Viobility™ 405/452 Fixable Dye (Miltenyi Biotech, Teterow, Germany) was used according to manufacturer’s protocol. Data were analyzed with FlowJo V10 software (Ashland, OR, USA). Moreover, based on the flow cytometry results, the numbers of NK and cytotoxic T cells as well as Treg were calculated as a percentage of all viable tumor-infiltrating leukocytes and T cells, respectively. Additionally, the ratio of cytotoxic T cells to Treg was assessed.

### 2.10. Statistics

For statistical analysis, SigmaPlot software (Version 13.0, Jandel Scientific Software, San Rafael, CA, USA) was used. First, the acquired data sets were tested for normal distribution. Based on the outcome, either the Mann–Whitney-U-test or Student *t*-test, if the assumption of normality was met, and analysis of variance (ANOVA) to compare more than two samples regarding the significance of a metric trait, were applied. All data are presented as mean ± SEM (standard error of the mean). Survival probabilities (event-free survival (EFS)) were estimated using the LogRank test, and multiple comparison was done with the Holm–Sidak method for post-hoc testing. A tumor volume of 300 mm^3^ was defined as an event. A *p* value of <0.05 (* *p* or # *p*) was considered significant, <0.01 (** *p*) very significant and <0.001 highly significant (*** *p*).

## 3. Results

### 3.1. FAP-IL-2v Effects on ADCC Mediated by DB against NB Cells

To investigate whether the immunocytokine FAP-IL-2v augments ADCC mediated by DB against NB cells, we used a calcein-AM-based cytotoxicity assay. Untreated leukocytes and leukocytes incubated with IL-2 instead of FAP-IL-2v served as controls.

**Figure 1:**
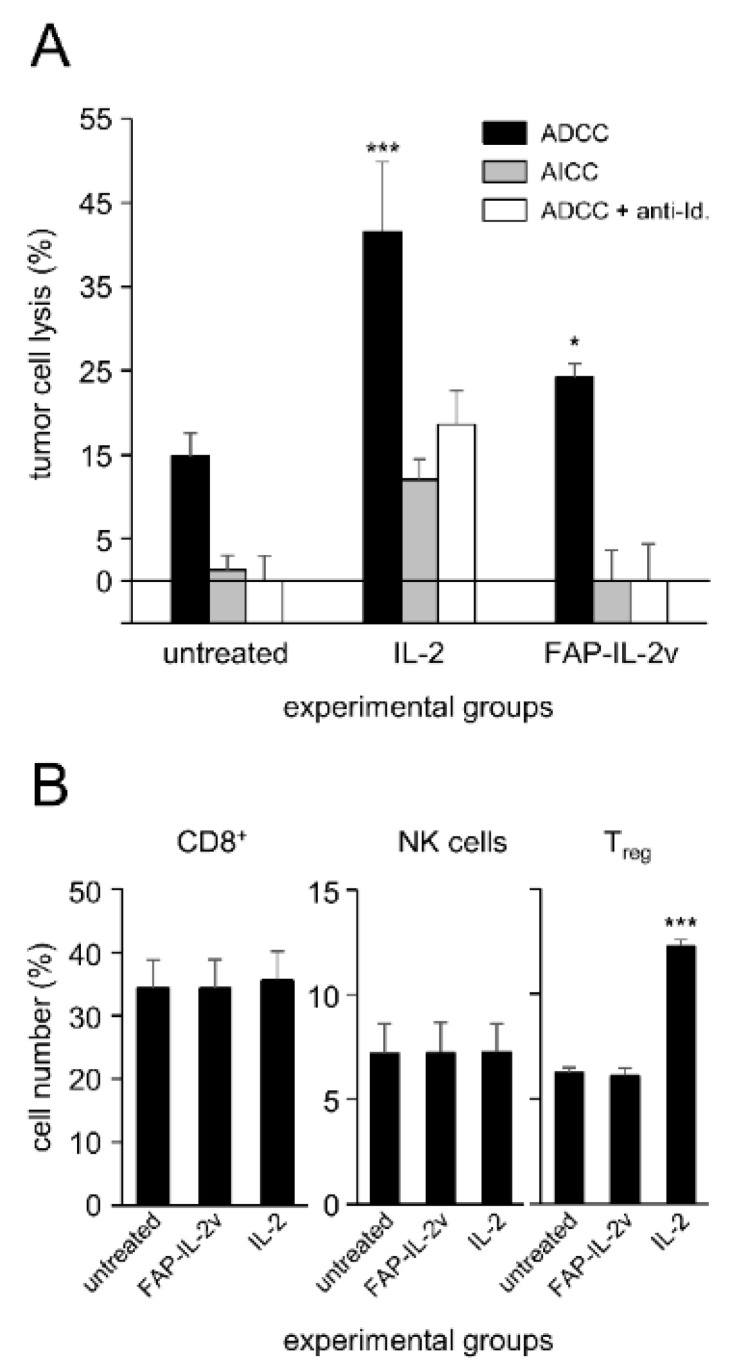
Analysis of FAP-IL-2v effects on GD_2_-directed ADCC mediated by dinutuximab beta and on the number of cytotoxic T cells, NK cells and Treg. Leukocytes of healthy donors were treated with either FAP-IL-2v or IL-2 for five days, followed by the analysis of the DB ADCC against the GD_2_-positive NB cells LAN-1 and flow cytometry analysis of different effector cell populations. Leukocytes cultivated without FAP-IL-2 and IL-2 served as negative controls (untreated). (**A**) ADCC (black columns) against tumor cells using DB and leukocytes treated with either FAP-IL-2 or IL-2 was assessed using a calcein-AM-based cytotoxicity assay. To show DB-independent tumor cells’ lysis (AICC; grey columns) and the GD_2_ specificity of the ADCC (white columns), additional samples were incubated with rituximab instead of DB and the anti-idiotype Ab of DB ganglidiomab, respectively. *t*-test. *** *p* < 0.001 vs. untreated ADCC; * *p* < 0.05 vs. untreated ADCC. (**B**) The effects of FAP-IL-2v and IL-2 on the number of cytotoxic T cells (CD8+), NK cells and Treg were analyzed using flow cytometry. Data are presented as % of the cells relative to all CD3+, lymphocytes and CD4+ cells for cytotoxic T cells, NK cells and Treg, respectively. ANOVA followed by appropriate post-hoc comparison test. *** *p* < 0.001 vs. untreated and FAP-IL-2v.

As expected, IL-2 treatment enhanced the cytotoxic activity of leukocytes against the GD_2_-positive NB cells LAN-1 (Figure 1A) compared to the untreated controls. This effect was GD_2_-specific, as incubation with the anti-idiotype Ab ganglidiomab completely abrogated ADCC, resulting in similar cytotoxicity levels compared to the negative control (AICC). Similar to the effects observed for IL-2, treatment of leukocytes with the immunocytokine FAP-IL-2v significantly increased ADCC mediated by DB (Figure 1A). An additional incubation with ganglidiomab confirmed the GD_2_ specificity of the observed effects.

These data clearly show a positive effect of FAP-IL-2v on ADCC against NB cells mediated by DB.

### 3.2. FAP-IL-2v and IL-2 Effects on Treg

Next, we investigated effects of FAP-IL-2v on Treg. Leukocytes incubated with IL-2 served as controls. Additionally, the effects of both agents on NK cells and cytotoxic T cells (CD8+) were evaluated.

As expected, incubation of leukocytes with IL-2 resulted in a strong increase in Treg numbers compared to the untreated controls (Figure 1B). In contrast, we found similar numbers of Treg after the treatment of leukocytes with FAP-IL-2 compared to the negative control, clearly confirming the fact that IL-2v does not stimulate this cell population. Interestingly, we did not observe any change in the CD8+- and NK cell numbers compared to the untreated controls (Figure 1B).

In summary, incubation of leukocytes with FAP-IL-2v did not stimulate Treg, in contrast to the strong effects of IL-2, leading to an almost two-fold increase in their numbers. These data suggest the rationale of using FAP-IL-2v in combination with DB instead of IL-2.

### 3.3. FAP Expression by Neuroblastoma Cells

To investigate FAP expression by human NB cells, both well-known (CHLA-15, CHLA-20, CHLA-90, CHLA-136, CHLA-172, LAN-1, Kelly and SMS-KCN) and primary cell lines established from tumor samples derived from high-risk NB patients (HGW-1, HGW-3, HGW-5 and HGW-B) were analyzed by RT-PCR.

**Figure 2:**
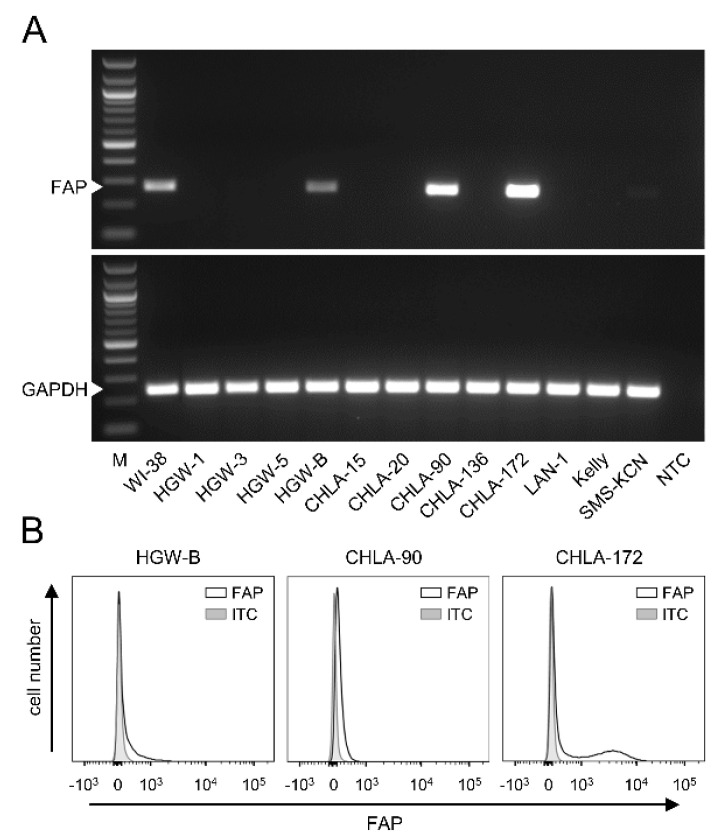
Analysis of FAP expression by human NB cells. (**A**) Representative images of the RT-PCR analysis of FAP mRNA levels (PCR product size: 268 bp) in NB cells derived from tumors of high-risk NB patients (HGW-1, HGW-3, HGW-5, HGW-B) and well-known NB cell lines (CHLA-15, CHLA-20, CHLA-90, CHLA-136, CHLA-172, LAN-1, Kelly, SMS-KCN). Human fibroblasts WI-38 were used as a positive and GAPDH (PCR product size: 238 bp) as an internal control. M: marker, NTC: no template control. (**B**) Representative histograms of the flow cytometry analysis of FAP abundance in human NB cells (HGW-B, CHLA-90, CHLA-172). Cells were stained with anti-human FAP-PE Ab (black curve) or appropriate ITC (grey-filled curve). Full gel images can be found at Supplementary Materials.

While most of the human cell lines analyzed were found to be FAP-negative, FAP mRNA was detected in the cell lines HGW-B, CHLA-90 and CHLA-172 (Figure 2A). Additional analysis of FAP surface abundance using flow cytometry confirmed our RT-PCR results, showing different levels of FAP for these three cell lines (Figure 2B).

These results show both FAP-positive and FAP-negative NB cells, suggesting FAP’s role in NB.

### 3.4. Impact of Tumor Cells on FAP Expression by Primary Fibroblasts (PAMF)

**Figure 3:**
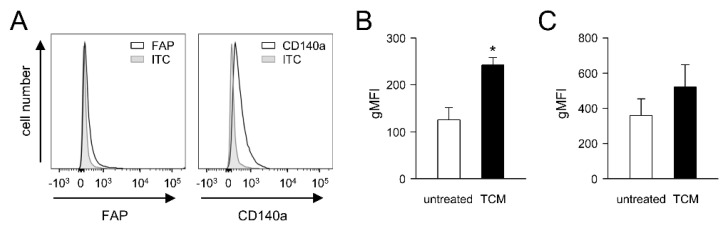
Analysis of tumor-cell-dependent effects on FAP abundance in PAMF. (**A**) Representative histograms of flow cytometry analysis of baseline levels of FAP and CD140a by PAMF. Primary fibroblasts were isolated from skin of female adult A/J mice and cultivated for up to two passages prior to analysis. Cells were then stained with mouse anti-FAP IgG and PE-labeled anti-murine IgG Ab, which served as primary and secondary Ab, respectively (black curve), and with FITC-labeled anti-mouse CD140a Ab or appropriate ITC (grey-filled curve). (**B**,**C**) Impact of soluble factors secreted by the tumor cells NXS2-HGW on FAP (**B**) and CD140a expression by PAMF (**C**). PAMF were incubated with either TCM (TCM, black columns) or control medium for 48 h (untreated, open columns). Expression levels are presented as gMFI quantified according to the following formula: gMFI of sample—gMFI of ITC. *t*-test. * *p* < 0.05.

Since soluble factors released by tumor cells can induce CAF development in tumor tissue [15,16], we investigated whether the expression of FAP by primary fibroblasts can be affected by the soluble factors released by the murine NB cells NXS2-HGW used in our in vivo experiments. We first isolated primary adult murine fibroblasts (PAMF) from the skin of adult A/J mice, followed by the flow cytometry analysis of the basal FAP abundance (Figure 3A), as described in the Materials and Methods Section. Additionally, a fibroblast marker, CD140a, was used to identify fibroblasts by flow cytometry.

As expected, PAMF showed a clear signal for CD140a (Figure 3A). Similarly, flow cytometry analysis revealed a basal level of FAP (Figure 3A), thus confirming the suitability of both markers for fibroblast detection. Importantly, incubation of PAMF with tumor-conditioned medium (TCM) for 48 h resulted in a significant, approximately two-fold increase in FAP expression compared to the untreated control (Figure 3B). Interestingly, a TCM-dependent increase in CD140a was also observed (Figure 3C); however, the difference was not statistically significant.

These results clearly show a tumor-cell-dependent enhancement in FAP levels by primary fibroblasts, suggesting the NB-dependent induction of CAFs in tumor tissue.

### 3.5. FAP Expression in Primary Murine Tumor Tissue

**Figure 4:**
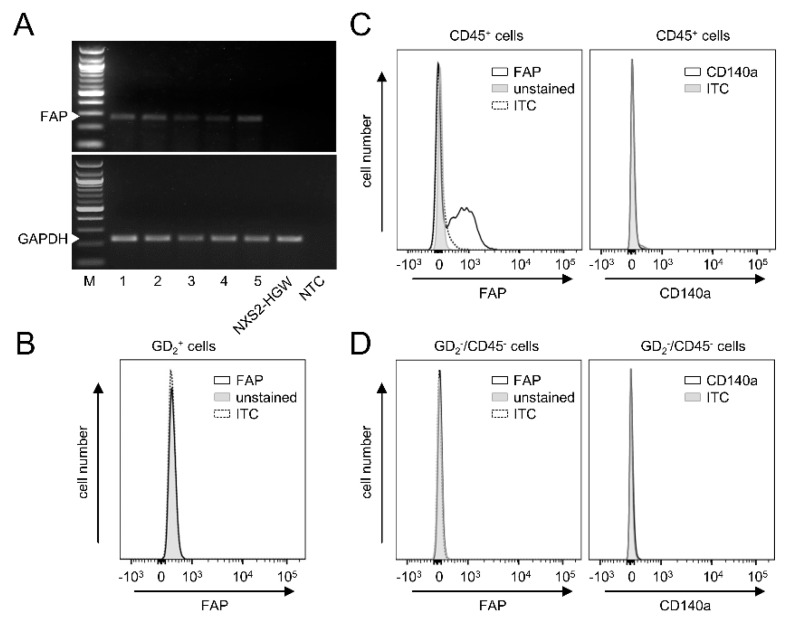
Analysis of FAP and CD140a expression in murine tumor tissue. (**A**) Representative images of the RT-PCR analysis of FAP mRNA levels (PCR product size: 268 bp) in five tumor tissue samples (1–5) and by the murine tumor cells NXS2-HGW that were used for tumor cell implantation. GAPDH (PCR product size: 238 bp) served as an internal control. M: marker, NTC: no template control. (**B**) A representative histogram of the flow cytometry analysis of FAP and CD140a abundance in the GD_2_- and CD45 double-negative cells within primary tumor tissue. Samples were collected when tumors reached a size of 750 mm^3^, followed by enzymatic digestion to obtain a single-cell solution. Samples were then stained with rabbit anti-mouse anti-FAP IgG and PE-labeled anti-rabbit IgG Ab, which served as primary and secondary Ab and FITC-labeled anti-mouse CD140a Ab, respectively (black curve), or appropriate ITC (black dashed curve). Unstained cells served as negative control (unstained; grey-filled curve). (**C**) A representative histogram of the flow cytometry analysis of FAP and CD140a abundance in the CD45-positive cells detected in tumor tissue. (**D**) A representative histogram of the flow cytometry analysis of FAP levels in NXS2-HGW cells that served for induction of primary tumors. Full gel images can be found at Supplementary Materials.

Next, we analyzed FAP mRNA levels in primary tumors collected from the experimental mice three weeks after s.c. injection of the syngeneic NB cells NXS2-HGW. RT-PCR analysis revealed a clear FAP signal in all tumor tissue samples analyzed (Figure 4A).

Interestingly, in contrast to the tumor tissue, we could not detect any FAP mRNA by the GD_2_-positive murine NB cells NXS2-HGW that served for tumor establishment in vivo (Figure 4A). These results could be confirmed by flow cytometry, showing a lack of FAP by NXS2-HGW (Figure 4D).

Next, we analyzed which cell populations within the tumor tissue express FAP. We defined CAFs as double-positive cells for FAP and CD140a and double-negative cells for GD_2_ (NB-specific marker) and CD45 (leukocyte-specific marker). Surprisingly, we could not detect CAFs in tumor tissue (Figure 4B), thus suggesting a lack of these cells in our model. Interestingly, FAP could be clearly detected by the CD45 cell population (Figure 4C), suggesting a role for FAP as a target in the tumor microenvironment expressed by tumor-infiltrating leukocytes.

In summary, our results show clear FAP expression in primary tumor tissue, thus showing the suitability of our syngeneic tumor model to test a combinatorial treatment with DB and the immunocytokine FAP-IL-2v. Since the NB cells that were used for tumor implantation were found to be FAP-negative and we could not detect CAFs in the primary tumor tissue, we hypothesized FAP’s role by tumor-infiltrating leukocytes.

**Figure 5:**
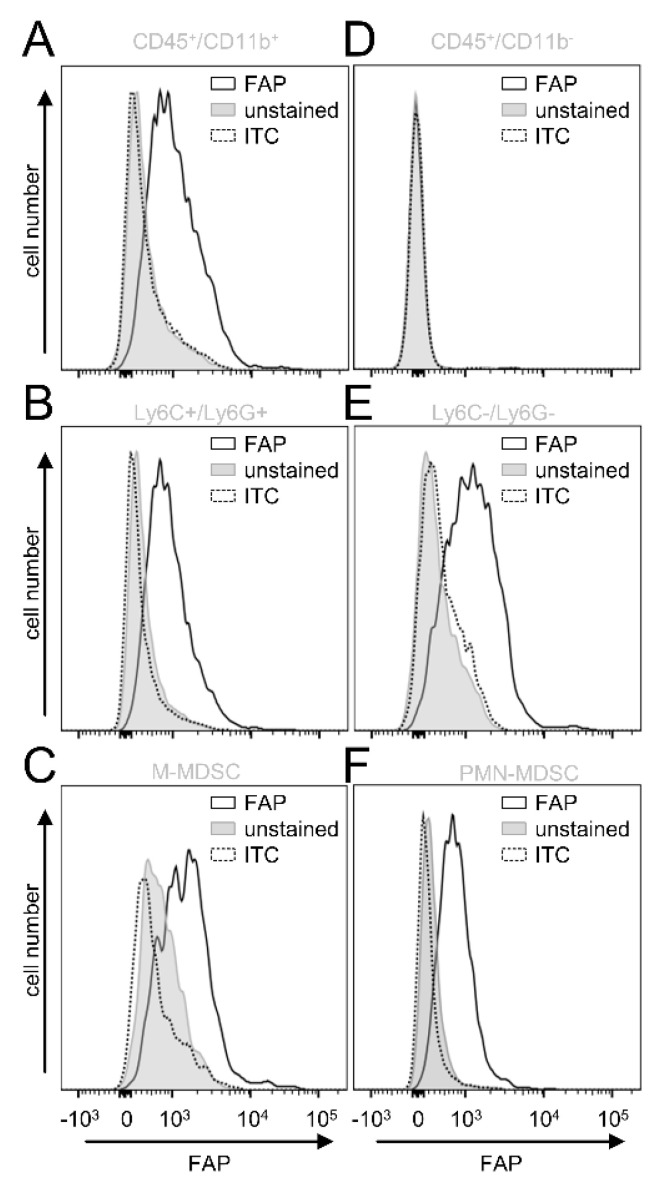
Analysis of FAP expression by tumor-infiltrating leukocytes. (**A**–**F**) Representative histograms of the flow cytometry analysis of FAP expression by leukocytes (CD45+) found within primary tumor tissue. Samples were collected when tumors reached a size of 750 mm^3^, followed by enzymatic digestion to obtain a single-cell solution. Samples were then analyzed to show FAP expression (black solid line) using mouse anti-FAP IgG and PE-labeled anti-murine IgG Ab as primary and secondary Ab by CD11b-positive (**A**; CD45+/CD11b+) and CD11b-negative leukocytes (**D**; CD45+/CD11b−), MDCS (**B**; CD45+/CD11+/Ly6C+/Ly6G+), CD11b+ leukocytes excepting MDSC (**E**; CD45+/CD11b+/Ly6C−/Ly6G−), as well as two MDCS populations, M-MDSC (**C**; CD45+/CD11b+/Ly6C^high^/Ly6G−) and PMN-MDCS (**F**; CD45+/CD11b+/Ly6C^low^/Ly6G+). Unstained cells (unstained; grey-filled curve) and cells incubated with appropriate isotype control (ITC, grey dashed line) served as negative controls.

Next, we investigated in more detail which cell populations of the tumor-infiltrating leukocytes express FAP. Since we previously showed a tumor-promoting role of CD11b-positive cells in NB [14], we assessed first FAP expression by this cell population (GD_2_-/CD45+/CD11b+). In contrast to the CD11b-negative cells (GD_2_−/CD45+/CD11b−) (Figure 5D), CD11b-positive cells showed a clear signal for FAP (Figure 5A), suggesting an additional role of CD11b-positive cells in FAP-mediated effects in NB.

To further characterize the FAP-positive CD11b cell fraction, we included additional cell markers to determine the immune-suppressive cells of the myeloid lineage MDSC, namely monocytic (M)- (CD11b+/Ly6C^high^/Ly6G−) and polymorphonuclear (PMN)-MDSC CD11b+/Ly6C^low^/Ly6G+). Interestingly, both MDSC populations (Figure 5C,F) and the CD11b+ cells that did not express Ly6C and Ly6G showed a clear FAP signal (Figure 5B,E).

In summary, analysis of tumor tissue revealed a lack of CAFs in our in vivo model. Importantly, we detected FAP on tumor-infiltrating CD11b+ cells, especially by M- and PMN-MDSC, as well as CD11b+ cells that did not express Ly6C and -G, probably tumor-associated macrophages (TAM), as has been shown in tumors of Lewis lung carcinoma [17].

### 3.6. Impact of Fibroblasts Injected in Combination with Tumor Cells on Tumor Growth

Based on our data showing the tumor-cell-dependent induction of FAP on primary fibroblasts and on the fact that CAFs were found in human NB [9,15], but not in our murine tumor model, we addressed the question of whether the injection of murine tumor cells in combination with PAMF results in the development of CAF-positive tumors. Tumor development was evaluated daily after the implantation of tumor cells in combination with PAMF in a ratio of 2:1 in comparison to the growth of tumor cells injected without PAMF.

**Figure 6:**
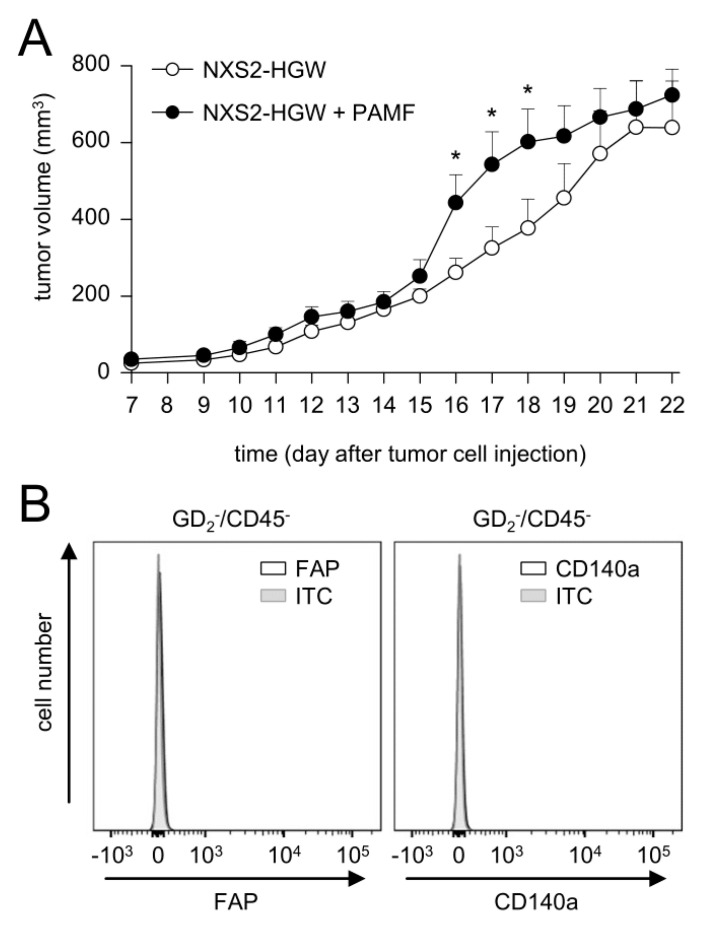
Impact of PAMF on tumor growth. (**A**) Analysis of tumor growth in mice injected with either NXS2-HGW (open circles) or NXS2-HGW in combination with PAMF (closed circles). Tumors were surgically resected on day 22 after tumor cell injection. Co-injection was performed at the tumor-cell-to-PAMF ratio of 2:1. When mice were sacrificed ahead of schedule due to tumor burden, the last measurement was included into the calculation of tumor growth at subsequent time points. Data are given as mean + SEM. * *p* < 0.05, *t*-test (**B**) Representative histograms of flow cytometry analysis of FAP and CD140a levels in tumor tissue of mice injected with NXS2-HGW in combination with PAMF. To detect CAFs (FAP+/CD140a+), leukocytes and tumor cells were excluded from the analysis using CD45 and GD_2_ expression, respectively.

Although the analysis of tumor growth revealed significantly higher tumor volumes in mice injected with tumor cells in combination with PAMF between days 16 and 18 compared to the controls (tumor cells only), tumor growth in both groups was found to be very similar on most days (Figure 6A). Unexpectedly, flow cytometry analysis of tumor tissue collected three weeks after the injection of tumor cells in combination with PAMF did not show any FAP- or CD140a-positive cells in the GD_2_−/CD45− cell population (Figure 6B).

These results indicate that the development of CAFs in our model could not be effectively induced by the co-injection of syngeneic primary fibroblasts and tumor cells. Based on these observations, we injected NB cells without PAMF for tumor induction in further in vivo experiments.

### 3.7. Establishment of a More Resistant In Vivo Tumor Model

Since the immunotherapy with DB (i.p., five consecutive days, 3 mg/kg bw/day, start of treatment: four days after tumor cell implantation) showed, in our syngeneic tumor model, strong antitumor efficacy against NB, resulting in constant tumor regression [12,14], we aimed to establish a more resistant version of this tumor model allowing the evaluation of the combinatorial immunotherapy with DB and FAP-IL-2v. For this, we started DB treatments in a later tumor growth phase (day 11, 12 and 14), after the development of measurable tumors.

**Figure 7:**
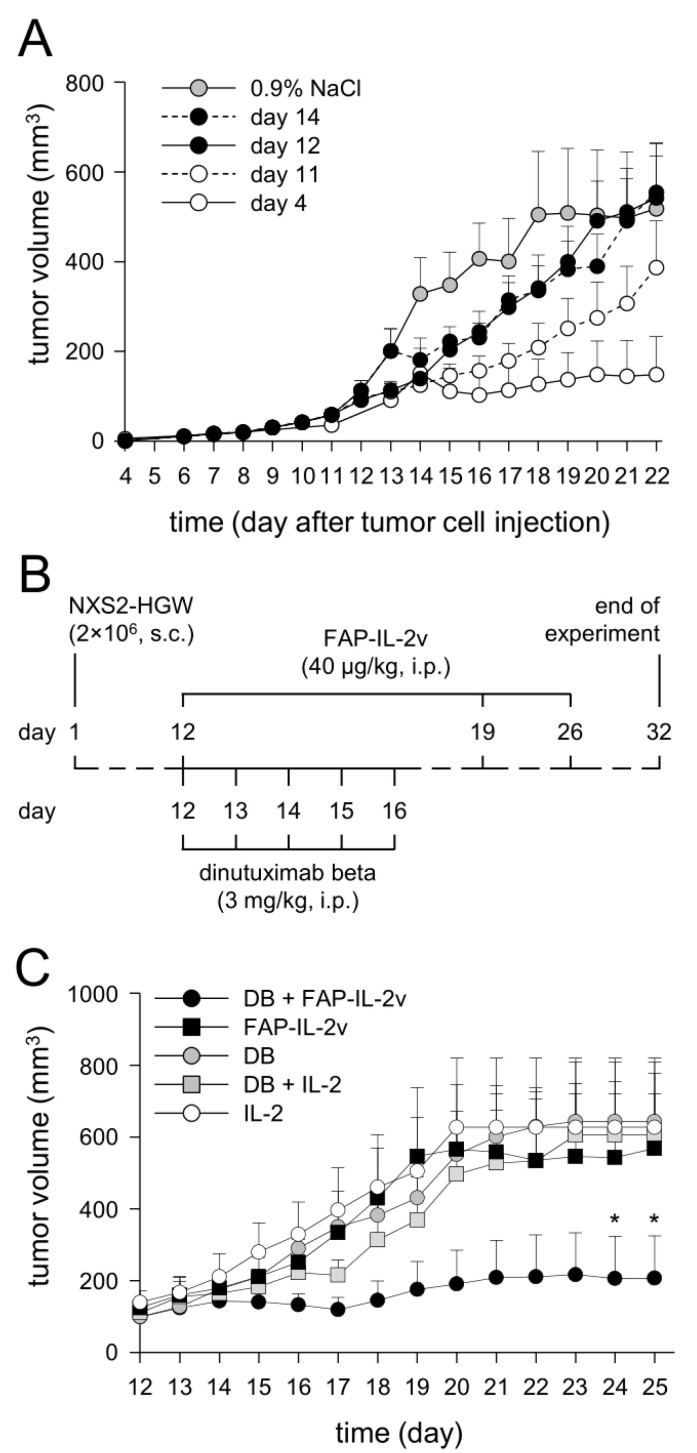

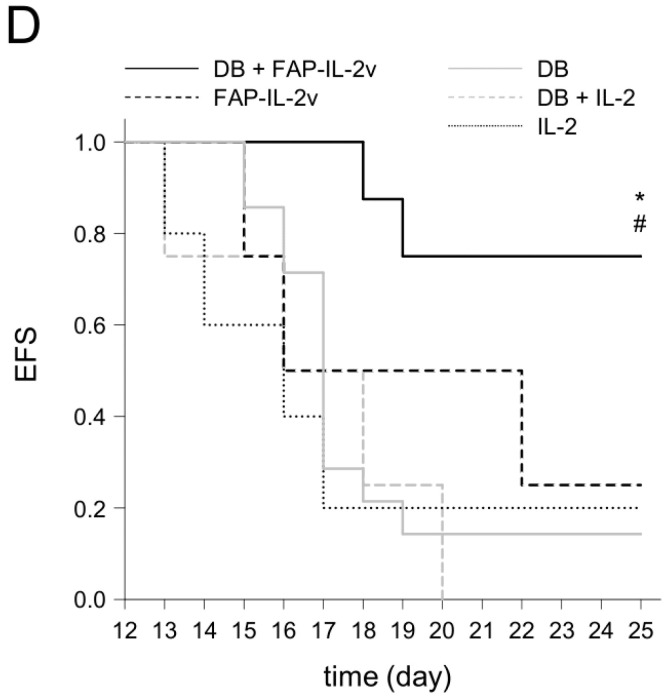
Establishment of a more resistant in vivo tumor model (**A**) and effects of the combinatorial immunotherapy with DB and FAP-IL-2v in vivo (**B**–**D**). (**A**) To establish a more resistant version of the murine syngeneic NB tumor model, DB treatment was started in a later tumor growth phase. After establishment of primary tumors, three later time points at which DB treatment was started (day 11 (open circles, dashed line), 12 (closed circles, solid line) and 14 (closed circles, dashed line)) were compared with day 4, representing the DB treatment starting time point of the previous NB model (open circles, solid line). Mice receiving equivalent doses of 0.9% NaCl served as controls (grey circles, solid line). When mice were sacrificed ahead of schedule due to tumor burden, the last measurement was included into the calculation of tumor growth at subsequent time points. Data are given as mean + SEM. (**B**) Schematic overview of the treatment protocol. The murine syngeneic GD_2_-expressing NB cells NXS2-HGW were injected on day 1, followed by establishment of primary tumors. When tumor size of 100 mm^3^ was reached (indicated as day 12), treatment was started. Mice received either DB or FAP-IL-2v or a combination of both. To investigate IL-2-dependent effects, mice of two additional control groups were treated with IL-2 and DB in combination with IL-2. Tumor growth was determined daily. (**C**) Analysis of tumor growth in mice treated with DB in combination with FAP-IL-2v (DB + FAP-IL-2v, black solid line, closed circles), FAP-IL-2v (black solid line, closed squares), DB (black solid line, grey circles), DB in combination with IL-2 (DB+ IL-2, black solid line, grey squares) and IL-2 (black solid line, open circles). When mice were sacrificed ahead of schedule due to tumor burden, the last measurement was included into the calculation of tumor growth at subsequent time points. Data are given as mean + SEM. * *p* < 0.05 vs. DB + IL-2, *t*-test. (**D**) Analysis of event-free survival (EFS) probabilities in mice treated with DB in combination with FAP-IL-2v (DB + FAP-IL-2v, black solid line), FAP-IL-2v (black dashed line), DB (grey solid line), DB in combination with IL-2 (DB+ IL-2, grey dashed line) and IL-2 (black dotted line). A tumor volume of 300 mm^3^ was defined as an event. Statistical analysis was performed using LogRank test; multiple comparison was done with Holm–Sidak method. * *p* < 0.05 vs. DB; # *p* < 0.05 vs. DB + IL-2.

As expected, starting the DB immunotherapy on days 11, 12 and 14 resulted in a steady decrease in the antitumor effects compared to starting on day 4 (Figure 7A). Untreated mice showed the strongest tumor growth compared to every experimental group receiving DB; however, the differences between the tumor volumes of the untreated mice and the mice of the two groups “day 12” and “day 14” were statistically not significant, thus indicating the development of a more resistant tumor against DB treatment compared with the mice of the “day 11” and “day 4” groups.

For further experiments, we used the treatment schedule of the first group of the treated mice that showed similar tumor growth to the untreated controls, namely the “day 12” group. In this group, tumors achieved a volume of approximately 100 mm3 at the start of treatment.

### 3.8. Evaluation of Antitumor Effects of Combinatorial Immunotherapy with DB and FAP-IL-2v

After the successful establishment of a more resistant version of our syngeneic tumor model allowing the evaluation of the antitumor efficacy of combinatorial therapeutic strategies, we treated mice showing tumors of approximately 100 mm^3^ volume with DB in combination with the immunocytokine FAP-IL-2v (Figure 7B). Additionally, mice treated with IL-2 instead of FAP-IL-2v, as well as mice receiving IL-2 only, served as controls.

As expected, the immunotherapy with DB showed, in the resistant tumor model, similar tumor growth compared to the monotherapy controls with FAP-IL-2 or IL-2 (Figure 7C). Interestingly, additional treatment of mice that received DB with IL-2 did not show any beneficial effects of IL-2 on tumor growth compared with the mice that were treated with DB without IL-2 (Figure 7C). In contrast, the combinatorial immunotherapy with DB and FAP-IL-2v resulted in superior antitumor effects, showing the strongest tumor growth inhibition compared to every control group (Figure 7C). These results clearly show that an additional treatment with FAP-IL-2v Ab augments the efficacy of the immunotherapy with DB against NB.

Further analysis of EFS confirmed our results of tumor growth evaluation. The superior effects on EFS could be observed in the mice treated with DB in combination with FAP-IL-2v (Figure 7D), further underlining the improvement in anti-GD_2_ Ab immunotherapies by the immunocytokine FAP-IL-2v against NB.

Together, our results show an FAP-IL-2v-dependent improvement in the DB-mediated antitumor effects against resistant NB, resulting in delayed tumor growth and an increase in survival compared to the respective monotherapy. Moreover, IL-2 did not show any benefit in combination with DB, confirming data reported in high-risk NB patients [4].

### 3.9. Assessment of Therapy-Dependent Effects on Tumor-Infiltrating Lymphocytes

Finally, we investigated the effects of the combinatorial treatment on different leukocyte populations infiltrating tumor tissue. We focused our analysis on the antitumoral effector NK and cytotoxic T cells (CD8+) as well as immune-suppressive Treg. Although the flow cytometry analysis was performed using tumors showing a volume of around 750 mm^3^, i.e., in a late growth phase, we still could observe clear therapy-dependent effects.

**Figure 8:**
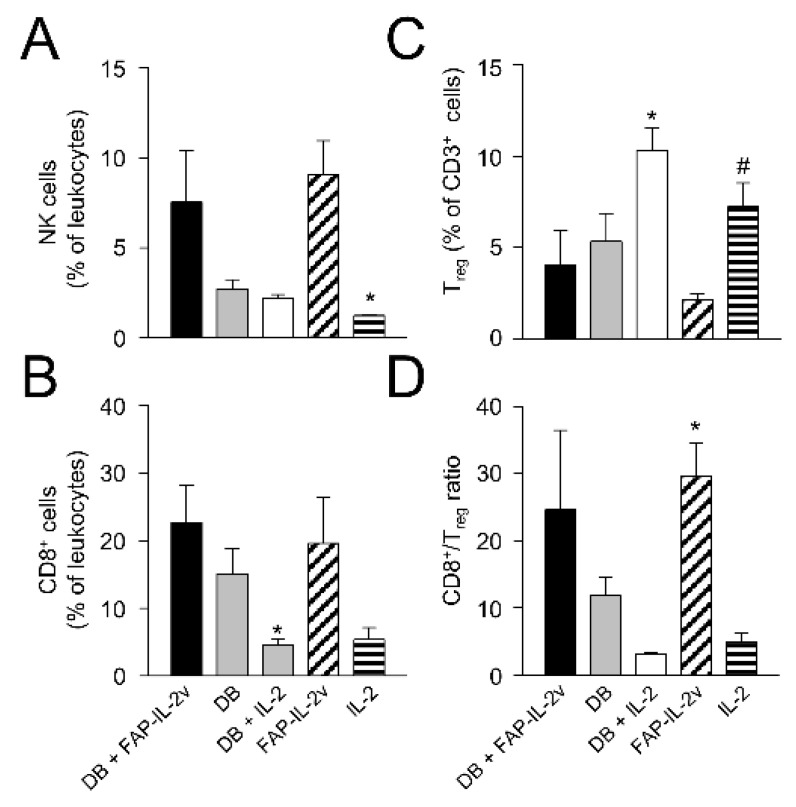
Flow cytometry analysis of tumor-infiltrating lymphocytes. To investigate effects of the combinatorial DB + FAP-IL-2v treatment on tumor-infiltrating lymphocytes, primary tumor tissue was analyzed using flow cytometry. After resection, primary tumor tissue samples were enzymatically digested to obtain a single-cell solution. To assess NK (**A**) and cytotoxic T cells (**B**) as well as Treg (**C**), the effector-cell-population-specific antigens CD335 and CD8 as well as CD25 and FocP3 were marked, respectively. Additionally, the ratio of cytotoxic T cells to Treg (**D**) was calculated. Results are presented as a percentage of the respective effector cell population cells relative to all viable CD45- or CD3-positive leukocytes for NK and cytotoxic T cells or Treg, respectively. ANOVA followed by appropriate post-hoc comparison test and *t*-test. (**A**) * *p* < 0.05 vs. FAP-IL-2v, (**B**) * *p* < 0.05 vs. DB + FAP-IL-2v, (**C**) * *p* < 0.05 vs. DB + FAP-IL-2v, # *p* < 0.05 vs. FAP-IL-2v, (**D**) * *p* < 0.05 vs. IL-2.

Compared to the control mice treated with IL-2 only, mice of the DB immunotherapy group showed higher numbers of NK and CD8+ T cells (Figure 8A,B), indicating the induction of antitumoral effector cells. However, the difference between the groups was statistically not significant, probably due to the low number of tumors available for the analysis. Furthermore, we observed, in the mice treated with DB, reduced Treg numbers (Figure 8C), thus further indicating the antitumor efficacy of the anti-GD_2_ Ab DB even in a more resistant model. As hypothesized, an additional treatment with the immunocytokine FAP-IL-2v augmented the antitumor effects of DB, resulting in a further increase in NK and CD8+ T cells as well as a reduction in Treg (Figure 8A–C). However, a single-agent treatment of mice with FAP-IL-2 Ab also led to elevated numbers of NK and CD8+ T cells as well as a reduction in Treg (Figure 8A–C), suggesting the immune-stimulating effects of IL-2v.

These results could be clearly confirmed by the analysis of the CD8+/Treg ratio, showing the highest levels in the mice receiving either DB in combination with FAP-IL-2v or FAP-IL-2v as a single-agent treatment (Figure 8D).

Together, the single-agent treatment of resistant NB with the anti-GD_2_ Ab DB resulted in an increase in antitumoral NK and CD8+ T cells as well as a reduction in immune-suppressive Treg-infiltrating primary tumors. The combinatorial treatment of mice with DB and FAP-IL-2v further increased the infiltration of tumors by NK and CD8+ T cells and resulted in a further reduction in Treg, probably due to the preferential stimulation of antitumoral effector cells by IL-2v. These results show an improvement in the DB immunotherapeutic efficacy by the immunocytokine FAP-IL-2, thus suggesting this combinatorial treatment as a promising strategy against resistant NB.

## 4. Discussion

The successful treatment of high-risk NB remains a major challenge in pediatric oncology. Although immunotherapeutic approaches, especially with monoclonal anti-GD_2_ Ab, have shown promising results, around one third of NB patients still die [18]. To improve the antitumor efficacy of anti-GD_2_ Ab, different cytokines were additionally included into the treatment protocols. The most prominent are IL-2 and GM-CSF, which activate two cell populations primarily mediating ADCC, namely NK cells and granulocytes, respectively. In Europe, an effective increase in NK cells could be shown in high-risk NB patients after the application of DB in combination with IL-2 compared to the patients of the IL-2-free treatment arm [5]. We here additionally confirmed in vitro a stimulating effect of IL-2 on the antitumor cytotoxicity of effector cells, showing an almost two-fold increase in ADCC mediated by DB after treatment of leukocytes with IL-2. However, the positive effects of IL-2 on NK cells and ADCC did not result in the improved survival of the high-risk NB patients compared to those patients who received immunotherapy without IL-2 [4]. A detailed comparison of the immune cells in the patients of both cohorts revealed a strong (21-fold) induction of Treg after application of IL-2 and almost unchanged Treg levels in the patients of the IL-2-free treatment arm [5]. Such a preferential induction of the immune-inhibiting cells by IL-2 can partly explain the missing survival benefit of the additional usage of IL-2 against NB, thus underlining a need for alternative strategies to activate antitumor effector cells only.

One promising alternative cytokine that showed the effective activation of NK cells, increasing the GD_2_-specific ADCC against NB cells in vitro, as well anti-NB efficacy in vivo, is IL-15 [19]. Importantly, recombinant human IL-15 has been already evaluated in cancer patients [20]. However, the systemic application of cytokines is associated with strong side effects. To overcome this problem, tumor-specific Ab conjugated with immune-stimulating cytokines, called immunocytokines, were developed and showed promising results in the treatment of cancer patients. In melanoma patients, treatment with the GD_2_-specitifc Ab hu14.18-IL-2 resulted in immune activation and showed reversible clinical toxicity with no grade 4 adverse events [21,22]. This immunocytokine was also used in clinical trials against refractory or recurrent NB, demonstrating safety profiles and antitumor efficacy [23,24]. Despite these promising results, the application of such immunocytokines can still activate Treg, thus hampering the antitumor effects of the immunotherapy.

Here, we investigated the anti-NB effects of the immunocytokine FAP-IL-2v in combination with DB. The rationale of using FAP-IL-2v was based on the fact that the mutated IL-2 (IL-2v) is able to preferentially stimulate antitumor effector cells such as NK cells without activating effects on the immune-inhibiting Treg [7]. Moreover, it was shown that the incubation of effector cells with FAP-IL-2v enhanced ADCC against colon and gastric cancer cells by the therapeutic Ab directed against tumor antigens [7]. In the present study, we could clearly confirm these results in NB, showing a strong FAP-IL-2-dependent increase in ADCC against tumor cells mediated by DB. Moreover, flow cytometry analysis of Treg did not show any induction compared to the untreated controls. In contrast, IL-2 treatment resulted in a strong increase in Treg, confirming the results in NB patients treated with DB in combination with IL-2 [5].

The observed increase in DB-specific ADCC by FAP-IL-2 in our in vitro experiments was clearly translated into an FAP-IL-2v-dependent improvement in the antitumor effects of DB against aggressively growing GD_2_-positive NB tumors in vivo. In contrast to our previously established tumor model [12], whereby treatments were started four days after tumor cell implantation, in the present study, we used a more resistant model of NB allowing the evaluation of combinatorial treatments. We changed the start of treatment to a later time point, at least 12 days after tumor cell implantation, after primary tumors were established at a size of 100 mm^3^, thus showing more resistant characteristics. Our in vivo data are in line with the data of Klein and colleagues, showing, in murine models of human cancers, such as leukemia, breast or lung cancer, FAP-IL-2v efficacy when combined with therapeutic Ab directed against tumor antigens [6]. Moreover, in the present study, the comparison of the antitumor efficacy of DB given as a monotherapy and DB in combination with IL-2 did not show any benefit regarding the additional treatment with IL-2, thus further confirming the results from the clinical study published by Ladenstein and colleagues showing a lack of superior effects of IL-2 [4].

Although the application of FAP-IL-2 has been reported to stimulate effector cells in the periphery [6] as well, the fusion of IL-2v to the anti-FAP Ab was performed to preferentially transport an additional stimulating agent (IL-2v) into the tumor tissue. Since FAP expression has been shown in NB and we could here confirm both FAP expression by human NB cell lines and in murine primary tumor tissue, as well as the NB-cell-dependent induction of FAP on fibroblasts, we suggest that both effects contribute to the efficacy of the immunocytokine FAP-IL-2v in the periphery and in the tumor tissue. In our tumor model, we detected FAP only by tumor-infiltrating CD11b-positive leukocytes, especially by the two MDCS populations, M- and PMN-MDSC. We found FAP also on the Ly6C and Ly6G double-negative cells, which are probably TAM, as has been reported by Arnold and colleagues [17]. To clarify the question of whether these FAP-positive cells in tumor tissue are indeed TAMs, further studies are required.

## 5. Conclusions

In summary, we showed a FAP-IL-2v-dependent increase in ADCC against NB cells mediated by the chimeric anti-GD_2_ Ab DB. We detected FAP in tumor tissue, with major expression by tumor-infiltrating MDSC. The combinatorial treatment of resistant NB with DB and FAP-IL-2 in vivo effectively inhibited tumor growth, improved the survival of tumor-bearing mice and resulted in an increase in cytotoxic T and NK cells, as well as a reduction in Treg found in tumor tissue. These data indicate that treatment with the immunocytokine FAP-IL-2v augments the efficacy of DB against resistant NB, probably by targeting MDSC and stimulating NK cells.

## Data Availability

The data of this study are available from the corresponding author upon reasonable request.

## Supplementary Materials

The following supporting information can be downloaded at: https://www.mdpi.com/article/10.3390/cancers14194842/s1. Full gel images can be found at Supplementary Materials.
