# Supplementary figures and images for: The Immunocytokine FAP-IL-2v Enhances Anti-Neuroblastoma Efficacy of the Anti-GD2 Antibody Dinutuximab Beta
Source: Cancers (Basel). 2022 Oct 4;14(19):4842. doi: 10.3390/cancers14194842 (PMC9563425; doi:10.3390/cancers14194842)

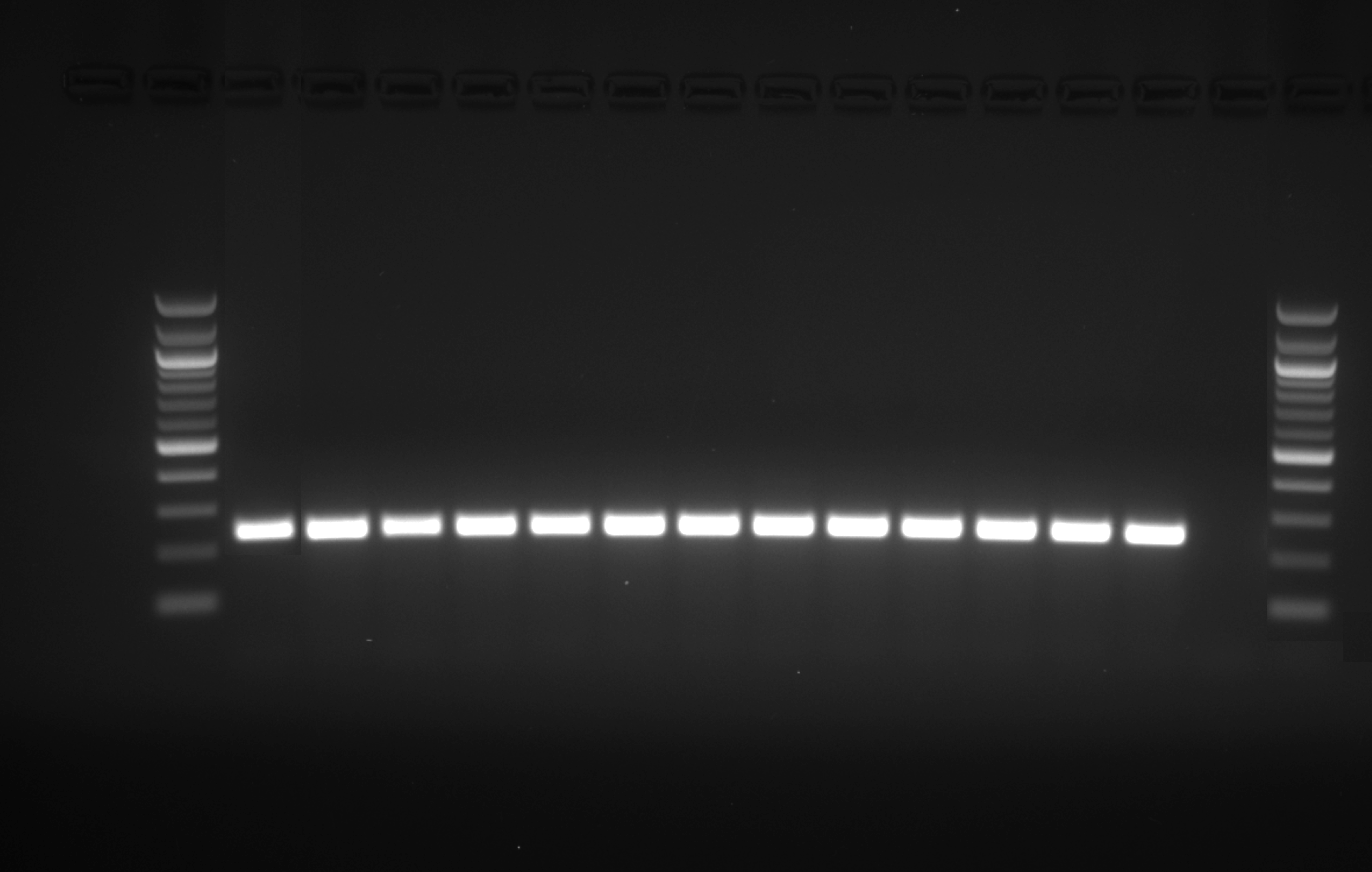

Supplement: Supplementary file 1 [file cancers-14-04842-s001.zip › 2022-02-22_mGAPDH_humane Zelllinien_NTC_1.tif]

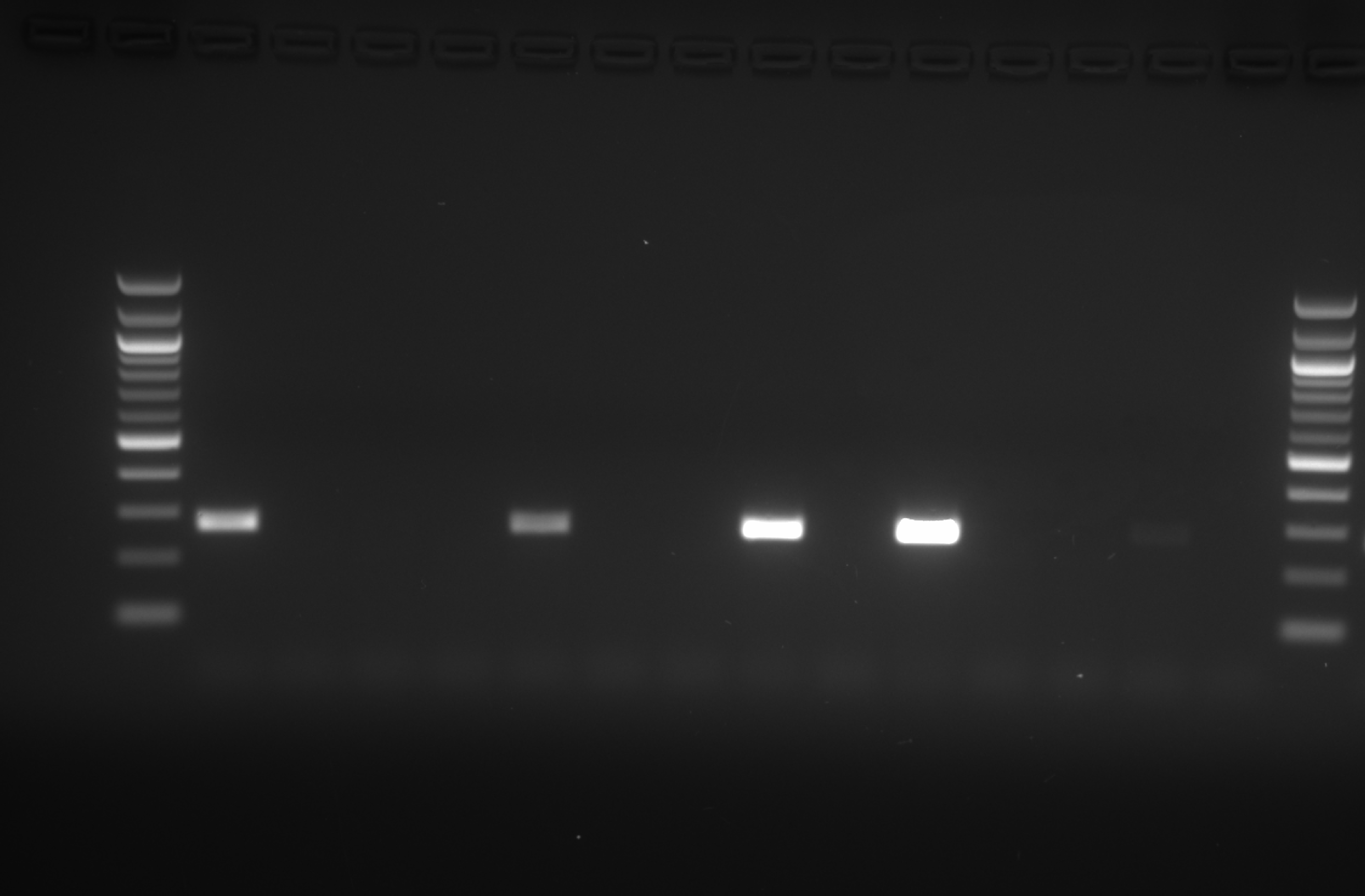

Supplement: Supplementary file 1 [file cancers-14-04842-s001.zip › 2022-02-22_m_h_FAP_humaneZelllinen_NTC_3.tif]

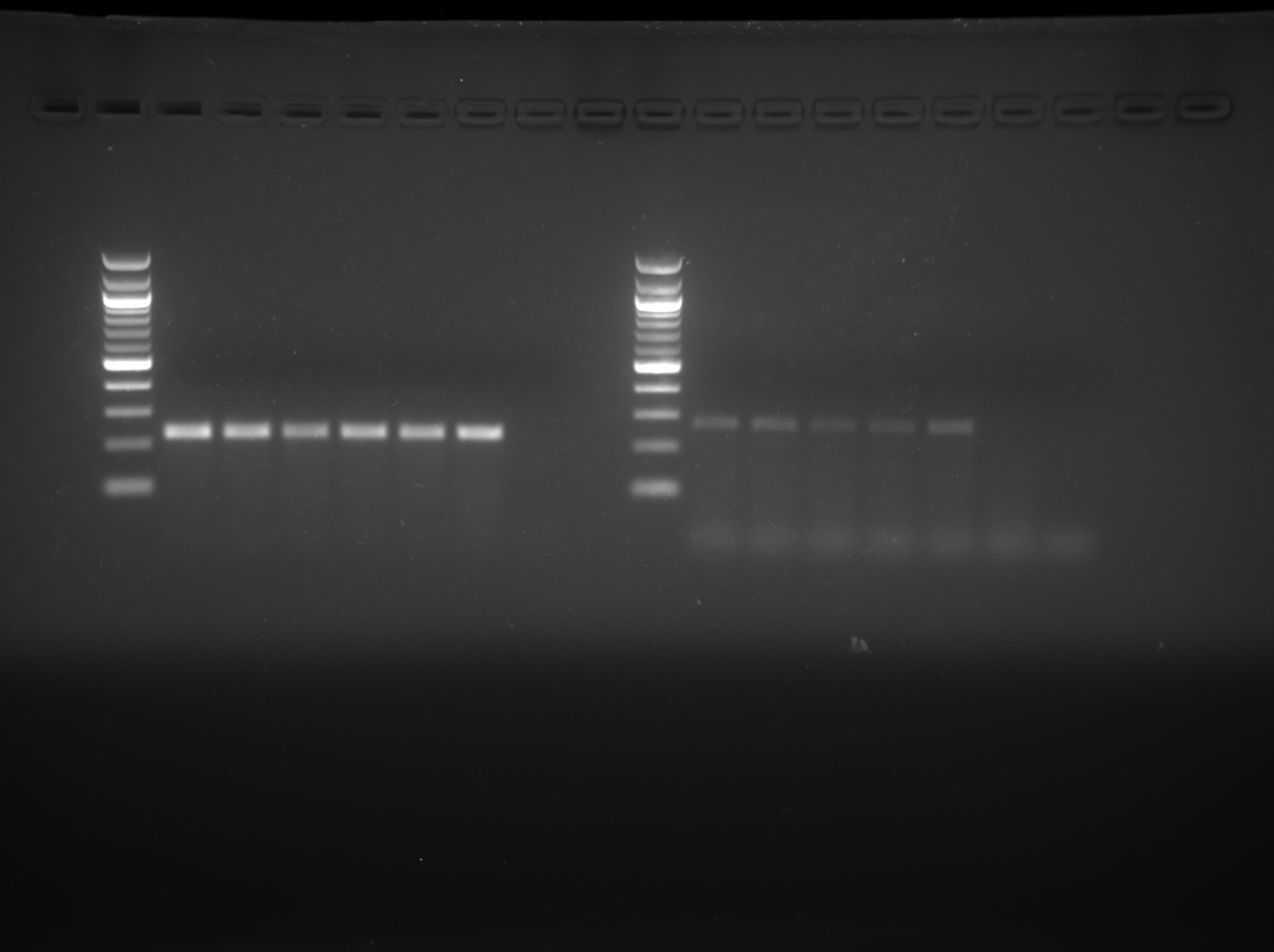

Supplement: Supplementary file 1 [file cancers-14-04842-s001.zip › RT-PCR_FAP_and_GAPDH_Fig._4A.tif]
